# Potential drug–drug interactions in the era of integrase strand transfer inhibitors: a cross-sectional single-center study in Japan

**DOI:** 10.1186/s40780-021-00226-7

**Published:** 2021-12-01

**Authors:** Yusuke Kunimoto, Ryosuke Matamura, Hiroshi Ikeda, Satoshi Fujii, Tomoko Kimyo, Manabu Kitagawa, Hiromasa Nakata, Masayoshi Kobune, Atsushi Miyamoto, Masahide Fukudo

**Affiliations:** 1 Department of Pharmacy, Sapporo Medical University Hospital, South-1, West-16, Chuo-ku, Sapporo, 060-8543 Japan; 2 Department of Hematology, Sapporo Medical University School of Medicine, South-1, West-16, Chuo-ku, Sapporo, 060-8543 Japan

**Keywords:** HIV, Potential drug–drug interaction, Antiretroviral therapy, Integrase strand transfer inhibitors, Polypharmacy

## Abstract

**Background:**

Potential drug–drug interactions (PDDIs) commonly occur because of aging and comorbidities in people living with human immunodeficiency virus (HIV; PLWH). Protease inhibitors and non-nucleoside reverse transcriptase inhibitors have been reported to cause PDDIs in these patients. However, there are few reports of PDDIs in the era of treatment using integrase strand transfer inhibitors. Therefore, we investigated PDDIs in Japanese PLWH receiving antiretroviral drugs (ARVs).

**Methods:**

This was a cross-sectional observational study conducted in Japanese outpatients. All eligible patients who had received ARV therapy for at least 48 weeks were enrolled. The primary endpoint was the incidence of PDDIs detected using the Lexicomp® interface.

**Results:**

Of the 71 eligible patients, 51 (71.8%) were prescribed concomitant non-ARV medications. In 21 patients (29.6%), PDDIs with the potential to reduce the effects of ARVs occurred, although the HIV load was suppressed in all cases. Polypharmacy (the use of ≥5 non-ARVs) was observed in 25 patients (35.2%). There was a significantly higher median number of non-ARV medications in the PDDI group than in the non-PDDI group (6 vs. 3, *P* <  0.001). Furthermore, the proportion of patients on polypharmacy was significantly higher in those with PDDIs than in those without PDDIs (81.0% vs. 26.7%, *P* <  0.001).

**Conclusions:**

The incidence of PDDIs is relatively high in Japanese PLWH, even in the era of treatment using integrase strand transfer inhibitors. Therefore, it is important for patients and health care providers to be constantly aware of PDDIs associated with ARV treatment.

**Supplementary Information:**

The online version contains supplementary material available at 10.1186/s40780-021-00226-7.

## Background

In recent years, the number of elderly people living with human immunodeficiency virus (HIV; PLWH) has been increasing, and problems related to comorbidities and concomitant drugs have garnered increasing attention [1, 2]. A previous report indicated that similar problems exist in Japan [3]. These problems associated with polypharmacy and potential drug–drug interactions (PDDIs) in PLWH have been noted to lead to adverse health outcomes [4]. Other reports have been dominated by PDDIs caused by protease inhibitors (PIs) and non-nucleoside reverse transcriptase inhibitors (NNRTIs), which inhibit or induce cytochrome P450 [5–7]. There are few reports of PDDIs in the era of treatment using integrase strand transfer inhibitors (INSTIs). Furthermore, the incidence of PDDIs in Japan and their negative effects are unknown. Trends for the prescription of antiretroviral drugs (ARVs) and non-ARVs vary by country. Therefore, local information is needed to effectively manage PDDIs. The purpose of this study was to investigate the incidence of PDDIs in medical settings in Japan.

## Methods

This cross-sectional observational study was conducted in Japanese outpatients aged 20 years or older who were prescribed ARVs between January 1, 2019, and April 30, 2019, in Sapporo Medical University Hospital. All eligible patients who had received antiretroviral therapy (ART) for at least 48 weeks were enrolled. Data from the patients’ last visit were used. The primary endpoint was the incidence of PDDIs detected using the Lexicomp® interface. We defined PDDIs that occurred between ARVs and non-ARVs as DDIs classified as Rank D (consider therapy modification) or Rank X (avoid combination) in the Lexicomp interface. Viral load suppression was defined as an HIV load of < 50 copies/mL, based on the FDA Snapshot Algorithm. The FDA Snapshot algorithm defines the success of ART by a viral load < 50 copies/mL. We calculated the proportion of patients with viral load suppression and compared clinical characteristics between patients with and without PDDIs. At the time of data collection, we did not analyze the changes in the ART regimen within a 48-week period before the visit. Concomitant medications were defined as medications received together for more than 2 weeks. Topical medications with the expectation of local action were not included. In addition, medications prescribed at other hospitals were not included in the analysis. Each component of fixed-dose combination medications (e.g., sulfamethoxazole and trimethoprim) was counted and analyzed separately. In contrast, herbal medicines and mixtures of probiotics were counted as one drug each even if they contained two or more ingredients. Polypharmacy was defined as the use of five or more medications [8]. ARVs were not included in the definition of polypharmacy. In the descriptive analysis, the chi-square or Fisher’s exact test was used to compare proportions and Mann–Whitney U test was used to compare quantitative variables. Predictors of the incidence of PDDIs were analyzed by logistic regression. Statistical significance was defined as a two-sided *P*-value of < 0.05. All analyses were performed using StatMate IV (ATMS Co., Ltd., Tokyo, Japan). The study was approved (312–161) by the ethical review board of Sapporo Medical University, Japan.

## Results

The characteristics of the study population are described in Table 1. Of the 71 eligible patients, 51 (71.8%) were prescribed non-ARVs. Overall, the most frequently recorded key drug class was INSTIs (83.1%), followed by NNRTIs (9.9%), INSTIs + PIs (5.6%), and PIs (1.4%). Polypharmacy (the use of ≥5 non-ARVs) was observed in 25 patients (35.2%). The most frequent classes of drugs prescribed concomitantly were those for acid-related disorders, psycholeptics, and vitamins (Table 2). Forty-four PDDIs were detected in 21 patients (29.6%). Regarding the breakdown of 44 PDDIs detected for the ARV class, the results were as follows: 36 PDDIs for INSTIs, 4 PDDIs for NNRTIs, 2 PDDIs for PIs, 1 PDDI for NRTIs, and 1 PDDI for cobicistat. Calcium, magnesium, and iron agents accounted for 90.9% of the total non-ARVs that caused PDDIs (Table 3). PDDIs that may reduce the efficacy of ARVs were detected in 21 cases with 40 PDDIs: INSTIs and NNRTIs accounted for 36 and 4 PDDIs, respectively. The physicians instructed nine of these 21 patients to maintain appropriate medication intervals (e.g., dolutegravir was to be taken after breakfast and magnesium agents after dinner). HIV load was less than 50 copies/mL in all 21 patients. There was a significantly higher median number of non-ARV medications in the PDDI group than in the non-PDDI group (6 vs. 3, *P* <  0.001) (Table 1). Furthermore, the proportion of patients with polypharmacy was significantly higher among those with PDDIs than in those without PDDIs (81.0% vs. 26.7%, P <  0.001). We also evaluated the effect of the number of non-ARVs and polypharmacy on the incidence of PDDIs by performing univariate analysis (simple logistic regression model). The results showed that the number of non-ARVs (OR = 1.52, 95% CI [1.16–1.99], *P* < 0.003) and polypharmacy (OR = 11.69, 95% CI [3.01–45.40], *P* < 0.001) were associated with the occurrence of PDDIs.

**Table 1:** Demographic characteristics of the study patients with HIV infection

	Overall study patients	Use of non-HIV medications			
		Total	With PDDI	Without PDDI	*p*-value
Number of patients	71	51	21	30	–
Age (years), median (range)	45 (21–79)	46 (21–79)	45 (21–79)	49.5 (28–72)	0.250
Male	67 (94.4)	47 (92.2)	19 (90.5)	28 (93.3)	1.000
Prior AIDS diagnosis, n (%)	21 (29.6)	15 (29.4)	6 (28.6)	9 (30.0)	1.000
Time since diagnosis HIV (years), median (range)	7 (1–29)	8 (1–20)	9 (1–17)	7 (1–20)	0.901
HCV co-infection, n (%)	0 (0)	0 (0)	0 (0)	0 (0)	–
HBV co-infection (HBs-antigen positive), n (%)	5 (7.0)	4 (7.8)	1 (4.8)	3 (10.0)	0.634
HIV-RNA < 50 copies/mL, n (%)	70 (98.6)	50 (98.0)	21 (100)	29 (96.7)	1.000
CD4 cell count, median (range)	506 (94–1843)	483 (94–1843)	477 (94–1843)	488.5 (228–1043)	0.213
Backbone drug					
TAF/FTC, n (%)	37 (52.1)	24 (47.1)	11 (52.4)	13 (43.3)	0.578
ABC/3TC, n (%)	29 (40.8)	22 (43.1)	8 (38.1)	14 (46.7)	0.578
TDF/FTC, n (%)	1 (1.4)	1 (2.0)	0 (0)	1 (3.3)	1.000
NRTI-sparing regimen, n (%)	4 (5.6)	4 (7.8)	2 (9.5)	2 (6.7)	1.000
Key drug class					
INSTI, n (%)	59 (83.1)	41 (80.4)	17 (81.0)	24 (80.0)	1.000
NNRTI, n (%)	7 (9.9)	5 (9.8)	2 (9.5)	3 (10.0)	1.000
PI, n (%)	1 (1.4)	1 (2.0)	0 (0)	1 (3.3)	1.000
INSTI and PI, n (%)	4 (5.6)	4 (7.8)	2 (9.5)	2 (6.7)	1.000
Number of non-HIV medications, median (range)	3 (0–14)	4 (1–14)	6 (2–14)	3 (1–13)	< 0.001
Use of ≥5 non-HIV medications, n (%)	25 (35.2)	25 (49.0)	17 (81.0)	8 (26.7)	< 0.001

*Abbreviations*: *3TC* Lamivudine, *ABC* Abacavir, *FTC* Emtricitabine, *INSTI* Integrase strand transfer inhibitor, *NNRTI* Non-nucleoside reverse transcriptase inhibitor, *NRTI* Nucleoside reverse transcriptase inhibitor, *PI* Protease inhibitor, *TAF* Tenofovir alafenamide fumarate

**Table 2:** Drugs concurrently prescribed with ARVs

Drug Class	Count
Drugs for acid-related disorders	39
Psycholeptics	30
Vitamins	27
Drugs for the treatment of bone diseases	22
Mineral supplements	20
Lipid-modifying agents	17
Antigout preparations	13
Calcium channel blockers	11
Agents acting on the renin-angiotensin system	9
Drugs for obstructive airway diseases	8
Antibacterials for systemic use	7
Antihistamines for systemic use	6
Drugs for constipation	6
Drugs used in diabetes	6
Anti-anemic preparations	5
Antidiarrheals, intestinal anti-inflammatory/anti-infective agents	5
Psychoanaleptics	5
Beta blockers	3
Diuretics	3
Others	27

*Abbreviations: ARV* Antiretroviral drug

**Table 3:** Prevalence of clinically significant drug interactions

Antiretroviral drug	Drug paired	Number of interactions, n	% of 44 PDDIs
Risk X (avoid combination)			
Raltegravir	Magnesium Carbonate	2	4.5%
Raltegravir	Magnesium Oxide	1	2.3%
Risk D (consider therapy modification)			
Dolutegravir	Calcium Carbonate	12	27.3%
Dolutegravir	Magnesium Carbonate	12	27.3%
Dolutegravir	Magnesium Oxide	3	6.8%
Raltegravir	Calcium Carbonate	2	4.5%
Rilpivirine	Calcium Carbonate	2	4.5%
Rilpivirine	Magnesium Carbonate	2	4.5%
Cobicistat	Rosuvastatin	1	2.3%
Darunavir	Nifedipine	1	2.3%
Elvitegravir	Calcium Carbonate	1	2.3%
Elvitegravir	Calcium L-Aspartate Hydrate ^a^	1	2.3%
Elvitegravir	Magnesium Carbonate	1	2.3%
Raltegravir	Ferric Citrate	1	2.3%
Ritonavir	Nifedipine	1	2.3%
Tenofovir alafenamide fumarate	Loxoprofen	1	2.3%

*Abbreviation*: *PDDI* Potential drug-drug interaction

^a^ Analyzed as polyvalent cation-containing products

## Discussion

The results of this study indicate the following. PDDIs are common in the Japanese population using INSTIs. In addition, PDDIs between INSTIs and polyvalent cations that could reduce the effects of ARVs occurred, although the HIV load was suppressed in all cases. The prevalence of PDDIs in our study (29.6%) was within the range reported in previous HIV cohort studies (17–45%) [5–7, 9–12]. In general, INSTIs are associated with a lower risk of PDDIs than other classes of drugs [9–11]. A study with no INSTI use reported a high incidence of PDDIs (40%) [5]; conversely, another study with high INSTI use (48%) reported a low incidence of PDDIs (17%) [9]. Furthermore, in a study with 52% use of INSTIs, the incidence of PDDIs was 21%, and the incidence associated with INSTIs accounted for only 2% [11]. On the contrary, in our study with even higher INSTI use (89%), the incidence of PDDIs was slightly higher at 29.6% (21 in 71 patients), and the incidence associated with INSTIs was 50.7% (36 PDDIs in 71 patients). Our results indicate a different trend from the results of previous studies. The patients in our study frequently took antacids and mineral supplements, which caused many PDDIs involving INSTIs. PLWH have numerous risk factors for osteoporosis, for example, tobacco use, alcohol abuse, vitamin D insufficiency, ART, duration of HIV infection, renal disease, and diabetes [13, 14]. In our study, we noted cases of PDDIs involving calcium agents used to treat osteoporosis in PLWH on INSTIs. In particular, the use of calcium and magnesium compounds with denosumab in osteoporosis treatment was a major cause of PDDIs in our patients. In contrast, in previous studies, the use of PIs and cardiovascular drugs was the major cause of PDDIs [5, 7]. PDDIs caused by PI inhibition of CYP3A are associated with the occurrence of adverse effects of concomitant medications; however, there were only two PDDIs (4.5% of 44 PDDIs) in this study. The results of this study differ from those of previous studies, in that the majority of the PDDIs in the study occurred between INSTIs and polyvalent cations.

The present results support the findings of previous drug-utilization studies demonstrating that the number of medications prescribed is associated with the occurrence of PDDIs [15–21]. Prescribing issues are common in PLWH due to the presence of age-related comorbidities, organ dysfunction, physiological changes, and long-term exposure to ARVs leading to a higher risk for PDDIs and inappropriate drug use [4]. Although PDDIs or inappropriate medications may not always result in adverse outcomes, medication reconciliation and medication review of drugs prescribed should be considered to mitigate potential PDDIs.

The results of our study are valuable in identifying new issues in HIV treatment with INSTIs in Japan. Furthermore, it is interesting that poor viral control was not reported in any of the patients with PDDIs between INSTIs and polyvalent cations. The effects of PDDIs involving INSTIs and polyvalent cations can be minimized by ensuring adequate intervals between the times at which these medications are taken [22]. Notably, a prior study indicated that PDDIs involving INSTIs and polyvalent cations increased the risk of virologic failure [23]. However, this study neither analyzed the intervals between the intake of INSTIs and polyvalent cations nor educated patients about PDDIs. In our study, the prescriptive dose spacing strategy was used in 42.9% of the cases. Additionally, we collaborated with HIV specialist pharmacists and nurses to routinely educate patients about PDDIs when using the combination of INSTIs and polyvalent cations. This strategy of patient education about PDDIs may have reduced the risk of virologic failure. In previous studies, pharmacist interventions have been reported to prevent serious PDDIs [7]. Therefore, we believe that pharmacist intervention should be implemented as a part of routine medical care. PDDIs may not always have clinical consequences in patients. However, we believe that identifying them is important because they may increase the risk of toxicity and loss of therapeutic effects.

There are several important limitations to this study. No conclusions regarding cause and effect can be drawn from this study due to its cross-sectional design. We were unable to analyze the duration of use of the concomitant medications that caused the PDDIs and medications prescribed in other hospitals. Additionally, our study included no information on the changes in the ART regimen within a 48-week period before the visit at the time of data collection. In the future, we will conduct a longitudinal study using the best possible medication history including prescriptions from other hospitals to address these issues. Another limitation of this study is the small number of participants. This limitation should be resolved in the future by performing a multicenter study. In addition, the study was conducted in a single center; thus, the results might be vulnerable to center bias, and it may be difficult to extrapolate them to other related settings, except for the part where most of our subjects chose ARVs from treatment guideline recommendations [24].

## Conclusions

In conclusion, the incidence of PDDIs involving INSTIs and non-ARVs is relatively high in Japanese PLWH. Many guidelines recommend the use of INSTIs; therefore, their use is expected to increase in the future. In addition, the risk of PDDIs among PLWH increases with aging as they use more gastrointestinal and osteoporotic drugs. We conclude that appropriate prescribing strategies and patient education regarding PDDIs are important to avoid virologic failure.

## Supplementary Information

**Additional file 1: Table S1.** Drug classes concurrently prescribed with ARVs.

## Data Availability

All data generated or analyzed during this study are included in this published article.

## Abbreviations

HIV: Human immunodeficiency virus
PLWH: People living with HIV
PDDI: Potential drug–drug interaction
ARV: Antiretroviral drug
PI: Protease inhibitor
NNRTI: Non-nucleoside reverse transcriptase inhibitor
INSTI: Integrase strand transfer inhibitor
