# Supplementary material for: Potential drug–drug interactions in the era of integrase strand transfer inhibitors: a cross-sectional single-center study in Japan
Source: J Pharm Health Care Sci. 2021 Dec 1;7:43. doi: 10.1186/s40780-021-00226-7 (PMC8638141; doi:10.1186/s40780-021-00226-7)
Supplement: Supplementary file 1 — Additional file 1: Table S1. Drug classes concurrently prescribed with ARVs. [file 40780_2021_226_MOESM1_ESM.docx]

Appendix Table S1 Drug classes concurrently prescribed with ARVs

| Class | Count |
| --- | --- |
| Drugs for acid-related disorders | 39 |
| Psycholeptics | 30 |
| Vitamins | 27 |
| Drugs for the treatment of bone diseases | 22 |
| Mineral supplements | 20 |
| Lipid-modifying agents | 17 |
| Antigout preparations | 13 |
| Calcium channel blockers | 11 |
| Agents acting on the renin‐angiotensin system | 9 |
| Drugs for obstructive airway diseases | 8 |
| Antibacterials for systemic use | 7 |
| Antihistamines for systemic use | 6 |
| Drugs for constipation | 6 |
| Drugs used in diabetes | 6 |
| Anti-anemic preparations | 5 |
| Antidiarrheals, intestinal anti-inflammatory/anti-infective agents | 5 |
| Psychoanaleptics | 5 |
| Beta blockers | 3 |
| Diuretics | 3 |
| Antiepileptics | 2 |
| Anti-inflammatory and antirheumatic products | 2 |
| Medication for bile and liver therapy | 2 |
| Cough and cold preparations | 2 |
| Digestives, incl. enzymes | 2 |
| Drugs for functional gastrointestinal disorders | 2 |
| Endocrine therapy | 2 |
| All other therapeutic products | 1 |
| Analgesics | 1 |
| Antiprotozoals | 1 |
| Antithrombotic agents | 1 |
| Blood substitutes and perfusion solutions | 1 |
| Corticosteroids for systemic use | 1 |
| Nasal preparations | 1 |
| Others | 6 |
|  |  |

ARV: antiretroviral drug
